# Optimization of UAV Spraying in Mountainous Nanguo Pear Orchards: Effects of Canopy Size and Operational Parameters on Droplet Deposition and Penetration

**DOI:** 10.3390/plants15172678

**Published:** 2026-08-31

**Authors:** Shuang Guo, Zhuangzhuang Li, Jianghui Luo, Yuzhou Liu, Suyuan Ma, Wanting Sun, Weixiang Yao

**Affiliations:** 1 School of Intelligent Science and Information Engineering, Shenyang University, Shenyang 110044, China; guos@syu.edu.cn; 2 College of Information and Electrical Engineering, Shenyang Agricultural University, National Digital Agriculture Regional Innovation Center (Northeast), Shenyang 110866, China; 2025240164@stu.syau.edu.cn (Z.L.); 2025240173@stu.syau.edu.cn (J.L.); 2024240168@stu.syau.edu.cn (Y.L.); 2025240094@stu.syau.edu.cn (S.M.); 2025188034@stu.syau.edu.cn (W.S.)

**Keywords:** canopy penetration, canopy size, droplet deposition, Nanguo pear orchard, plant protection UAVs

## Abstract

The use of uniform spray volume rates for fruit trees with different canopy sizes is common in orchard spraying with plant protection unmanned aerial vehicles (UAVs), whereas the applicability of Leaf Wall Area (LWA)- and Tree Row Volume (TRV)-based methods to UAV spraying remains insufficiently validated. This study evaluated canopy size-based spray volume optimization and droplet deposition and penetration along the vertical canopy profile in a mountainous Nanguo pear orchard. The results showed that, under a uniform spray volume rate, small canopy trees exhibited significantly higher droplet deposition and ground deposition than large canopy trees, indicating greater potential spray losses. LWA- and TRV-based adjustment reduced the spray volume rate for small canopy trees by 43.0% and 49.0%, respectively, while maintaining comparable deposition in the upper and middle canopy layers. However, deposition in the lower canopy and on abaxial leaf surfaces remained limited, indicating that conventional LWA and TRV methods do not fully account for the top-down deposition characteristics of UAV spraying. Along the vertical canopy profile, finer atomization levels generally favored droplet penetration into the lower canopy, whereas increasing the spray volume rate increased overall deposition but did not significantly improve vertical penetration. Flight speed showed no consistent effect on penetration within the tested range. The results highlight canopy size as a key factor in UAV spray deposition. Canopy size-based variable-rate application can reduce spray volume rate while maintaining effective deposition, but further optimization should consider rotor-induced airflow and canopy structure.

## 1. Introduction

Nanguo pear (*Pyrus ussuriensis* Maxim.) is a distinctive pear cultivar widely grown in the hilly and mountainous regions of Haicheng, Liaoning Province, China. Due to the complex terrain and orchard conditions, pest and disease management remains a major challenge [1]. Ground-based sprayers are often unable to access these orchards, whereas manual spraying has low efficiency, high labor costs, and increased risks of pesticide exposure to operators. Furthermore, the ongoing shortage of agricultural labor caused by urbanization and population aging in China has further constrained the sustainable development of the Nanguo pear industry, highlighting the urgent need for more efficient spraying technologies [2,3].

Over the past decade, plant protection unmanned aerial vehicles (UAVs) have been rapidly adopted in China. Initially used mainly on field crops, UAV spraying has gradually expanded to orchards as the technology has matured and the field crop market has become increasingly saturated. Compared with field crops, orchard spraying is considerably more challenging because of the complex three-dimensional canopy architecture and heterogeneous operating environment [4,5]. Consequently, UAV spraying has emerged as a promising supplement to conventional ground-based and manual spraying for pest and disease control in mountainous orchards [6]. Previous studies have demonstrated the feasibility of applying UAVs in Nanguo pear orchards for plant protection operations [7,8].

The ultimate objective of UAV spraying is to deliver atomized droplets onto target crops through the interaction between the spray system and the rotor downwash airflow. However, droplet deposition is strongly influenced by crop growth characteristics and canopy morphology. Unlike field crops such as rice and wheat, which generally exhibit relatively uniform plant architecture, fruit trees are typical three-dimensional crops with substantial tree-to-tree variability, particularly in canopy size [9]. Consequently, orchard spraying requires more precise droplet deposition than field crop applications [10]. Under a constant spray volume, trees with different canopy sizes may receive substantially different amounts of spray. Large canopy trees may suffer from insufficient deposition and reduced pest control efficacy, whereas small canopy trees may experience excessive deposition, increasing the risks of phytotoxicity and pesticide loss [11]. Therefore, accurately evaluating droplet deposition among trees with different canopy sizes is essential for precision orchard spraying.

Previous studies have suggested that spray volume should be adjusted according to canopy characteristics rather than maintained at a constant level [12,13]. In ground-based orchard spraying, canopy-dependent spray volume adjustment has received considerable attention. Qiu et al. [14] compared five spray volume rates for large, medium, and small canopy fruit trees using an air-assisted sprayer and recommended optimal spray volume rates of 0.25, 0.20, and 0.14 L per tree, respectively. Van Zyl et al. [15] reported that canopy density was a critical factor affecting droplet deposition in citrus orchards sprayed with a tower sprayer. Garcerá et al. [16] further demonstrated that deposition differences among citrus trees increased with canopy size variability when using both knapsack and axial-fan sprayers. More recently, Liu et al. [17] developed a LiDAR-based variable-rate spraying system capable of adjusting spray volume in real time according to canopy volume, thereby reducing pesticide consumption while maintaining adequate spray coverage. Nevertheless, the influence of canopy size on UAV spray deposition has received relatively little attention. Moreover, two widely adopted canopy-based dose adjustment methods, Leaf Wall Area (LWA) and Tree Row Volume (TRV), have been extensively investigated for ground sprayers [18,19,20], whereas their applicability to UAV spraying has received limited systematic evaluation.

Accurate assessment of droplet deposition is equally important for evaluating spray performance and improving pesticide use efficiency in orchards [21]. Reliable deposition measurements provide quantitative information on spray distribution within the canopy, ensuring adequate coverage of target areas while maximizing pesticide utilization and pest control efficacy [22]. Furthermore, detailed deposition assessments can identify potential operational problems, such as uneven spray distribution, pesticide waste, and environmental contamination, thereby providing guidance for optimizing spraying parameters and application strategies [23,24].

Effective orchard spraying requires droplets to penetrate and deposit uniformly throughout the canopy. However, the upper canopy inevitably intercepts part of the downward-moving droplets generated by UAV spraying, making canopy penetration a critical concern for both growers and researchers [25]. Existing studies typically divide the canopy into three vertical layers to evaluate droplet deposition and penetration [26,27]. For Nanguo pear trees, whose canopy thickness is approximately 3 m, the vertical distribution of droplets, particularly within the middle and lower canopy, is crucial for effective pest control. However, recent research has shown that the conventional three-layer sampling strategy cannot adequately characterize the complex droplet distribution within Nanguo pear canopies, potentially leading to biased evaluations of spray performance [28]. Moreover, few studies have adopted finer vertical stratification to investigate droplet penetration in detail. Increasing the number of sampling layers is therefore the most direct approach to improve the accuracy and reliability of canopy deposition assessment by providing a more comprehensive description of droplet distribution throughout the canopy profile.

In addition, droplet deposition characteristics are strongly affected by orchard environment, canopy architecture, terrain, and operational parameters [29,30]. Consequently, conclusions derived from one orchard system cannot be directly transferred to another, and no universal recommendation can be applied across different orchard conditions [31]. In particular, studies on UAV spraying in mountainous Nanguo pear orchards remain limited, while investigations focusing on the canopy size effect are even scarcer [32]. These knowledge gaps highlight the necessity of conducting field experiments under representative orchard conditions.

Therefore, this study investigated UAV spraying in mountainous Nanguo pear orchards through two complementary experiments: canopy size-based spray volume optimization and droplet deposition evaluation, and the characterization of droplet deposition and penetration along the vertical canopy profile under different operational conditions. Specifically, droplet deposition characteristics were quantitatively compared between trees with two representative canopy sizes under both a constant spray volume and canopy-adjusted spray volumes determined using the LWA and TRV methods. Furthermore, a multi-layer canopy sampling strategy was employed to characterize vertical droplet penetration under different atomization levels and spray volume rates. The findings provide a theoretical basis and technical support for precision variable-rate UAV spraying in mountainous orchards.

## 2. Results and Discussion

### 2.1. Experiment I: Canopy Size-Based Spray Volume Optimization and Droplet Deposition Evaluation

#### 2.1.1. Droplet Deposition on the Adaxial Leaf Surfaces and Ground

**Figure 1:**
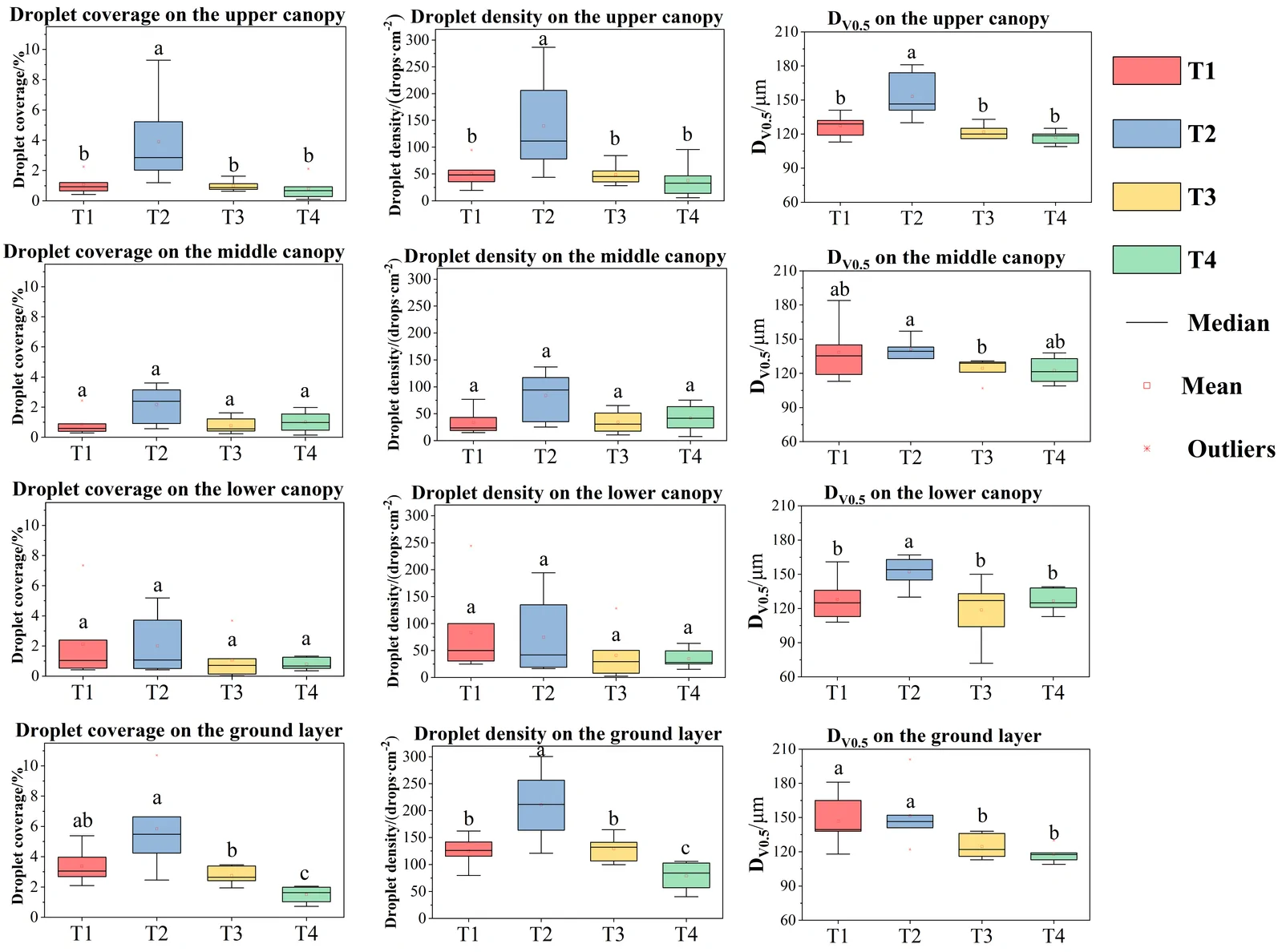
Droplet coverage, droplet density, and D_V0.5_ on adaxial leaf surfaces and the ground under different treatments. T1: large canopy trees with 60.0 L·ha^−1^; T2: small canopy trees with 60.0 L·ha^−1^; T3: small canopy trees with LWA-based adjustment (34.2 L·ha^−1^); T4: small canopy trees with TRV-based adjustment (30.6 L·ha^−1^). The same letters indicate no significant differences, whereas different letters indicate significant differences (*p* < 0.05).

Figure 1 presents the droplet deposition characteristics on the adaxial leaf surfaces of the upper, middle, and lower canopy layers, as well as on the ground beneath the canopy. The evaluated parameters include droplet coverage, droplet density, and D_V0.5_.

(1) Comparison of droplet deposition between different canopy sizes under the same spray volume rate

As shown in Figure 1, when the same spray volume rate (60.0 L·ha^−1^) was applied to trees with different canopy sizes, the small canopy trees (canopy size B) generally exhibited higher droplet coverage than the large canopy trees (canopy size A) across different sampling layers. A comparison between T1 and T2 revealed that the average droplet coverage in the upper canopy layer of T2 was 3.91%, which was significantly higher than that of T1 (1.07%) (*p* < 0.05), representing approximately 3.65 times that of T1. In the middle canopy layer, the average droplet coverage of T2 was 2.49 times higher than that of T1. However, in the lower canopy layer, the coverage values of the two treatments were relatively similar, with average values of 2.13% for T1 and 2.00% for T2. The variation pattern of droplet density was generally consistent with that of droplet coverage. In the upper canopy layer, T2 showed a significantly higher average droplet density (139.5 drops·cm^−2^) than T1 (50.5 drops·cm^−2^). Although the droplet density of T2 was also higher than that of T1 in the middle canopy layer, the difference was not statistically significant (*p* > 0.05). Interestingly, in the lower canopy layer, T1 exhibited a slightly higher average droplet density than T2.

Droplet size is another important indicator for evaluating spray deposition performance. For T1, the D_V0.5_ values in the upper, middle, and lower canopy layers were 127, 139, and 128 μm, respectively. In comparison, the corresponding D_V0.5_ values for T2 were 153, 141, and 152 μm, respectively. Statistical analysis showed that the differences in droplet size between T1 and T2 were significant in the upper and lower canopy layers (*p* < 0.05), whereas no significant difference was observed in the middle layer (*p* > 0.05).

The higher deposition observed in small canopy trees may be attributed to differences in canopy structure. The EA-30X UAV used in this study was equipped with CCMS-L20000 twin-peak atomizing nozzles, which generate fine droplets. Due to the relatively sparse canopy structure of canopy size B trees, smaller droplets were more easily transported by the rotor-induced airflow and penetrated into the canopy, resulting in higher droplet coverage and density in T2. When excessive droplets accumulated on the leaf surface, droplet overlap occurred on the WSPs, which may have led to an overestimation of measured droplet size [33].

Overall, under the same spray volume rate, small canopy trees received higher droplet deposition than large canopy trees, which was closely associated with canopy structural differences. Compared with large canopy trees, small canopy trees generally had younger growth characteristics, fewer branches, and a more open canopy structure. Therefore, droplets were more likely to penetrate through branches and foliage and reach different canopy positions. However, enhanced penetration in small canopy trees also resulted in greater droplet losses to the ground. At the ground sampling positions beneath the canopy, the droplet coverage and density of T1 were 3.37% and 125.2 drops·cm^−2^, respectively, whereas those of T2 reached 5.83% and 210.9 drops·cm^−2^, respectively. Similarly, the ground-layer D_V0.5_ of T2 (152 μm) was slightly higher than that of T1 (147 μm). These results confirmed that applying the same spray volume to smaller canopy trees resulted in greater spray losses to the ground. Ground deposition not only represents inefficient pesticide utilization but may also increase potential environmental risks associated with pesticide contamination [34]. Therefore, adjusting spray volume rate according to canopy size is necessary for UAV-based precision orchard spraying.

These findings are generally consistent with previous studies indicating that canopy structure is an important determinant of spray interception and deposition [22]. However, the present results further demonstrate the practical consequence of this relationship under UAV spraying in mountainous orchards: applying a uniform spray volume to trees with substantially different canopy sizes can result in markedly different deposition efficiencies and ground losses. In conventional orchard spraying, spray is generally introduced laterally into the canopy, whereas UAV spraying in this study was conducted from above the canopy [12,13]. Consequently, canopy size and openness directly affected the extent to which droplets were intercepted by foliage before reaching the ground. This suggests that canopy-size-dependent spray-volume adjustment is particularly important for UAV-based spraying in orchards with heterogeneous tree structures.

It should also be noted that the differences observed between the two canopy-size classes cannot necessarily be attributed to canopy size alone. Other structural characteristics, including tree height, canopy thickness, tree age, branch density, and canopy density, may also influence droplet transport and interception [35]. Therefore, the present comparison should be interpreted as an evaluation of two practically distinct canopy-size conditions rather than as an isolated causal effect of canopy size. Further studies using more quantitatively characterized canopy structures would be useful for separating the individual effects of these structural variables.

(2) Droplet deposition performance of canopy-size-adjusted spray volume rates based on LWA and TRV

For treatments T3 and T4, the spray volume rates for small canopy trees (canopy size B) were determined based on the LWA and TRV methods, respectively, using the spray volume rate of T1 as the reference. The measured results showed that the average droplet coverage values of T3 and T4 in the upper canopy layer were 0.99% and 0.80%, respectively. The corresponding droplet densities were 49.0 and 37.9 drops·cm^−2^, and the D_V0.5_ values were 122 and 117 μm, respectively. Compared with T4, T3 generally exhibited slightly higher droplet deposition in the upper canopy layer. A similar trend was observed in the lower canopy layer, whereas in the middle canopy layer, T4 showed slightly higher deposition than T3. Compared with T2, which used the same small canopy trees but received the conventional spray volume rate (60.0 L·ha^−1^), both T3 and T4 resulted in significantly reduced droplet deposition in the upper canopy layer (*p* < 0.05). Although droplet deposition in the middle and lower canopy layers was also lower in T3 and T4 than in T2, the differences were not statistically significant (*p* > 0.05). When compared with the large canopy reference treatment T1, T3 and T4 showed similar droplet deposition levels in the upper and middle canopy layers. However, deposition in the lower canopy layer was lower for both T3 and T4. Ground deposition was also detected in T3 and T4, but it was generally reduced compared with T1 and T2, which was mainly attributed to the reduced spray volume rates applied in these treatments.

The results indicated that when the spray volume rate for the large canopy trees was set at 60.0 L ha^−1^, adjusting the spray volume for the small canopy trees to 34.2 L·ha^−1^ and 30.6 L·ha^−1^ based on the LWA and TRV methods, respectively, resulted in comparable droplet deposition in the upper and middle canopy layers between the two canopy sizes. However, droplet deposition in the lower canopy layer was relatively low when the spray volume was determined using either the LWA or TRV method. This phenomenon may be related to the flight and spraying configuration of the UAV, as the downward transport of spray droplets is influenced by the canopy structure, with this effect being particularly pronounced in the lower canopy layer. A literature review further revealed that existing spraying studies based on the LWA or TRV methods have generally been conducted using ground-based sprayers. During ground-based spraying, the sprayer applies spray from the inter-row space toward both sides of the tree canopy, allowing droplets to enter the upper, middle, and lower canopy layers simultaneously from both sides. Consequently, similar deposition levels can be achieved across the different canopy layers [36]. In contrast, during UAV spraying, droplets are released from above the canopy and penetrate downward from the canopy top toward the bottom, passing through the upper layer first, followed by the middle layer, and finally reaching the lower layer. During this process, a large proportion of the droplets is intercepted by the upper and middle canopy layers, thereby reducing the number of droplets reaching the lower layer and consequently affecting droplet deposition in the lower canopy [37].

The comparison with previous LWA- and TRV-based studies helps to clarify the applicability and limitations of canopy-based spray-volume estimation methods for UAV orchard spraying. The results indicate that conventional canopy-based spray-volume estimation methods cannot be directly transferred to UAV applications without considering the differences in spray-entry geometry. Rather, these methods can provide a useful basis for adjusting spray volume according to canopy size, while their effectiveness is constrained by the fundamentally different spray-entry geometry of UAVs [38]. In the present study, both LWA- and TRV-based adjustments substantially reduced the spray volume applied to small canopy trees while maintaining deposition levels comparable with the large canopy reference in the upper and middle layers. However, neither method adequately compensated for the reduced deposition in the lower canopy. This indicates that canopy-based indices alone cannot fully characterize the vertical deposition process associated with top-down UAV spraying.

Overall, adjusting spray volume according to canopy size is necessary for UAV-based orchard spraying. In this study, LWA- and TRV-based spray volume estimation reduced the spray volume applied to small canopy trees while maintaining comparable droplet deposition in the upper and middle canopy layers, although additional optimization is required to improve lower canopy deposition [28,39]. Comparison between the two methods showed that LWA-based adjustment resulted in relatively higher deposition in the upper and lower canopy layers, whereas TRV-based adjustment produced slightly higher deposition in the middle canopy layer. Taken together, these results indicate that spray-volume optimization and canopy deposition cannot be considered independently in UAV orchard spraying. Adjusting spray volume according to canopy size can reduce excessive application and ground losses, but the resulting vertical deposition pattern remains constrained by canopy structure and the top-down spray trajectory. This distinction should be considered when adapting spray-volume concepts originally developed for conventional ground-based orchard spraying to UAV applications.

#### 2.1.2. Droplet Deposition on Abaxial Leaf Surfaces

**Figure 2:**
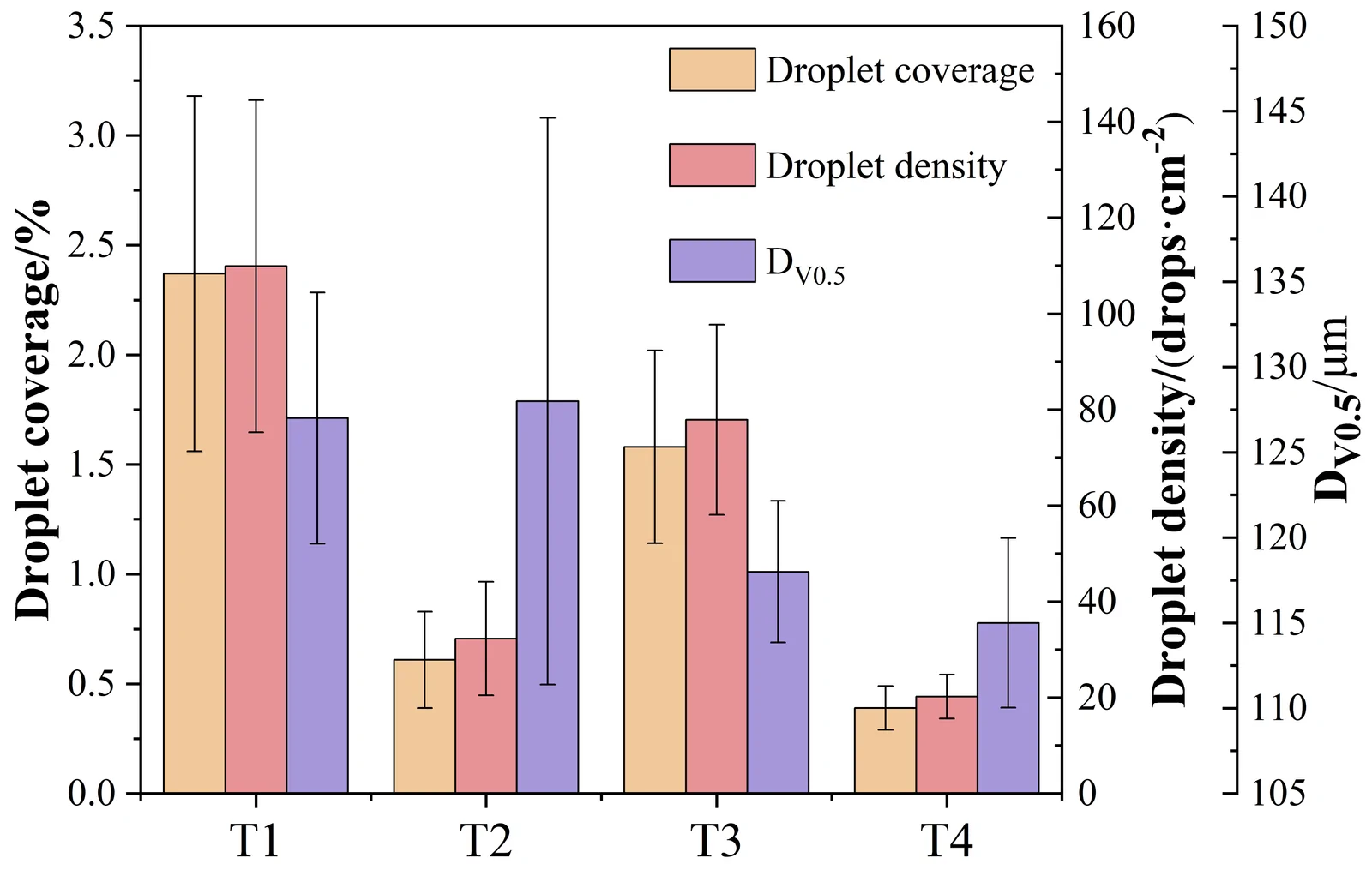
Comparison of droplet deposition on abaxial leaf surfaces among treatments.

Droplet deposition on the abaxial leaf surfaces represents another limitation affecting target coverage in UAV-based orchard spraying. The deposition characteristics on the lower leaf surfaces within the canopy were also evaluated for all treatments (Figure 2). Compared with the adaxial leaf surfaces, droplet deposition on the abaxial surfaces was generally lower, with greater variability among sampling positions. Among the four treatments, T1 exhibited the highest average droplet coverage and droplet density on the abaxial leaf surfaces, with values of 2.37% and 109.9 drops·cm^−2^, respectively. The highest D_V0.5_ value (128 μm) was observed in T2, whereas T4 showed the lowest values for all deposition parameters, which was consistent with its lower spray volume rate. Overall, similar to previous studies [7,40], droplet deposition on abaxial leaf surfaces was lower than that on adaxial surfaces. In addition, substantial variability was observed among sampling positions on the abaxial surfaces, indicating that droplet deposition on the lower leaf surface was more random. The relatively large standard errors shown in Figure 2 further supported this observation.

The detailed deposition parameters for the abaxial leaf surfaces at different canopy layers are summarized in Table 1. The droplet coverage, droplet density, and D_V0.5_ ranged from 0.11% to 5.13%, 4.3 to 227.1 drops·cm^−2^, and 104 to 143 μm, respectively. Among all treatments and sampling layers, the highest abaxial deposition was observed in the lower canopy layer of T1. The relatively high abaxial deposition observed in the lower canopy of T1 may be associated with the structural characteristics of large canopy trees. Due to artificial pruning management, some leaves located in the lower canopy had relatively sparse canopy structures above them. The reduced canopy obstruction may have allowed stronger interaction with rotor-induced airflow, causing leaf movement and increasing the probability of droplet interception on the abaxial surfaces [41]. Furthermore, the distribution of droplets on abaxial leaf surfaces across different canopy layers did not show a consistent pattern among treatments. This irregular distribution further supports the previous observation that droplet deposition on the abaxial surface is highly variable and difficult to predict under UAV spraying conditions.

The lower and more variable abaxial deposition observed in this study is also consistent with the inherent limitation of top-down UAV spraying. Although rotor-induced airflow and leaf movement may occasionally facilitate deposition on abaxial surfaces, such effects are strongly dependent on local canopy structure and leaf orientation [1]. Therefore, improving total canopy deposition through spray-volume adjustment does not necessarily guarantee improved deposition on the abaxial surface. This further supports the need to consider canopy structure and airflow characteristics when evaluating the biological coverage potential of UAV spraying, rather than relying solely on total droplet deposition.

It should be noted that the present study evaluated droplet deposition using water and water-sensitive paper rather than commercial pesticide formulations. While this approach provides a practical means of quantifying deposition and comparing treatments under controlled experimental conditions, formulation-dependent properties such as surface tension, viscosity, and evaporation characteristics may influence droplet transport, retention, and spreading under actual pesticide application conditions [42]. Therefore, the deposition patterns observed here should be interpreted primarily as a characterization of physical spray deposition rather than as a direct measure of pesticide efficacy.

### 2.2. Experiment II: Droplet Deposition and Penetration Characteristics Along the Vertical Canopy Profile

#### 2.2.1. Droplet Deposition in Different Canopy Layers

**Figure 3:**
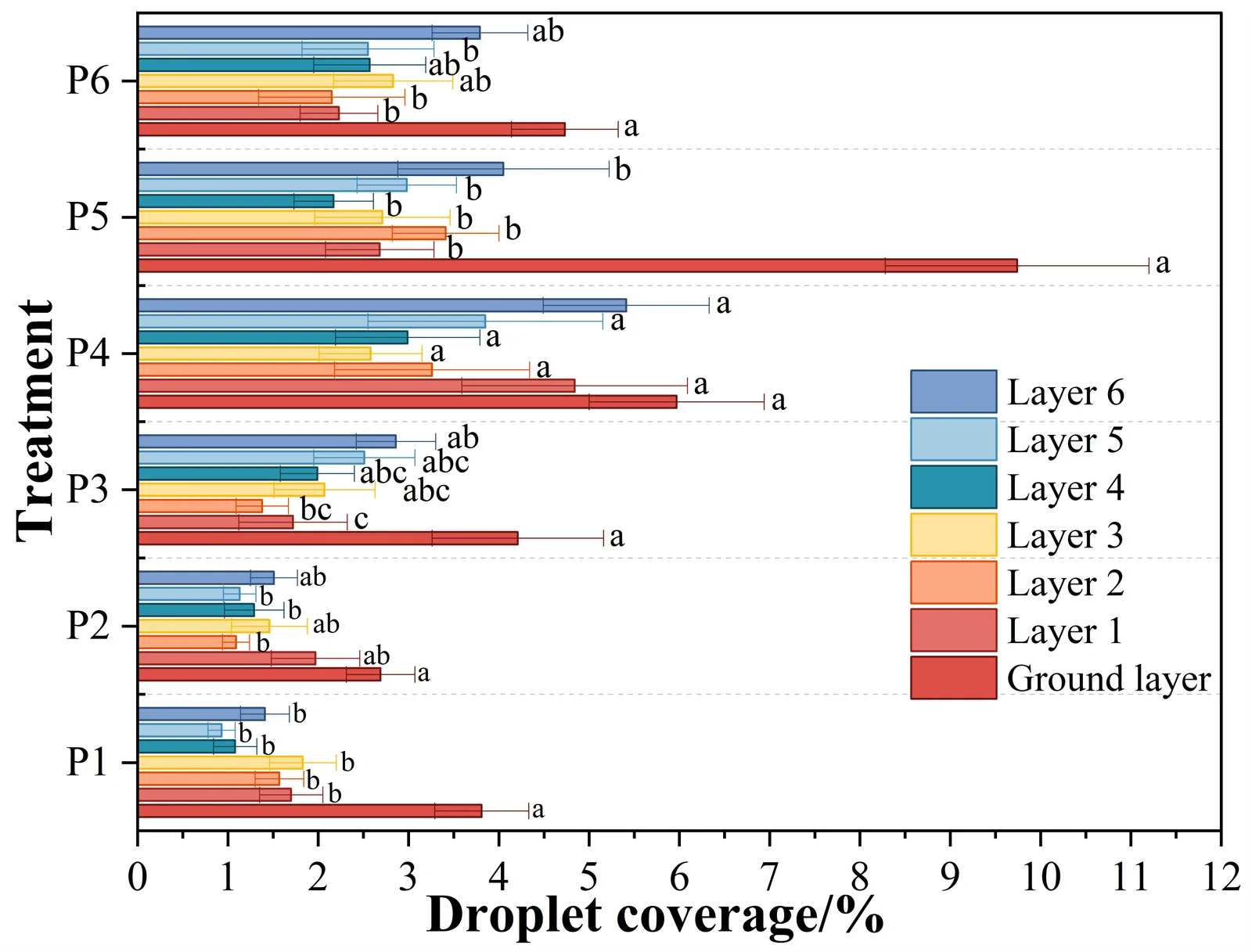
Droplet coverage across different layers of the vertical canopy profile under different treatments. The same letters indicate no significant differences, whereas different letters indicate significant differences (*p* < 0.05).

The vertical distribution of droplet coverage across the canopy layers for treatments P1-P6 is presented in Figure 3. Owing to differences in the experimental treatments, the droplet coverage varied among treatments. Within the canopy, droplet coverage ranged from 0.93% to 1.83% for P1, with the maximum value observed in Layer 3; from 1.09% to 1.97% for P2, with the maximum in Layer 1; from 1.38% to 2.86% for P3, with the maximum in Layer 6; from 2.58% to 5.41% for P4, also peaking in Layer 6; from 2.17% to 4.05% for P5, with the highest value in Layer 6; and from 2.15% to 3.79% for P6, again with the maximum in Layer 6. These results indicate that the canopy top (Layer 6) generally received the highest droplet deposition, which is consistent with the downward transport characteristics of UAV spraying. Among all treatments, P4 exhibited the largest variation in droplet coverage across the canopy layers, with a range of 2.83%. This was likely associated with its relatively high spray volume rate (90 L·ha^−1^), which resulted in greater differences in deposition among canopy layers. Considerable differences in droplet coverage were also observed at the ground sampling positions, where values ranged from 2.69% to 9.74%. The highest ground coverage was recorded in P5, indicating that a substantial proportion of droplets penetrated through the canopy and reached the ground. Considering the operational parameters, this result was most likely related to the relatively low flight speed of P5 (1.8 m·s^−1^). A lower flight speed increased the residence time of the UAV above the canopy, allowing droplets to interact with the canopy for a longer period. Under the influence of gravity and rotor-induced airflow, more droplets penetrated through the canopy and were ultimately deposited on the ground [43].

Statistical analysis further showed significant differences among sampling layers for most treatments, primarily between the canopy layers and the ground layer, where droplet deposition was generally higher. An exception was observed for P4, in which no significant differences were detected among the sampling layers. This result may be attributed to its higher spray volume rate (90 L·ha^−1^), which produced relatively high droplet deposition throughout the canopy and consequently reduced the differences among layers.

The overall vertical deposition pattern is consistent with the top-down spray trajectory of UAVs and with the general observation in previous UAV spraying studies that the upper canopy is more readily exposed to spray droplets than deeper canopy positions [7,44]. However, the present experiment further shows that the magnitude of this vertical difference depended on the combination of spray volume, atomization level, and flight speed. Thus, the vertical deposition pattern may be influenced not only by canopy position but also the interaction among droplet characteristics, spray volume, atomization level, and flight speed, which influence the time and airflow available for droplet transport through the canopy.

**Figure 4:**
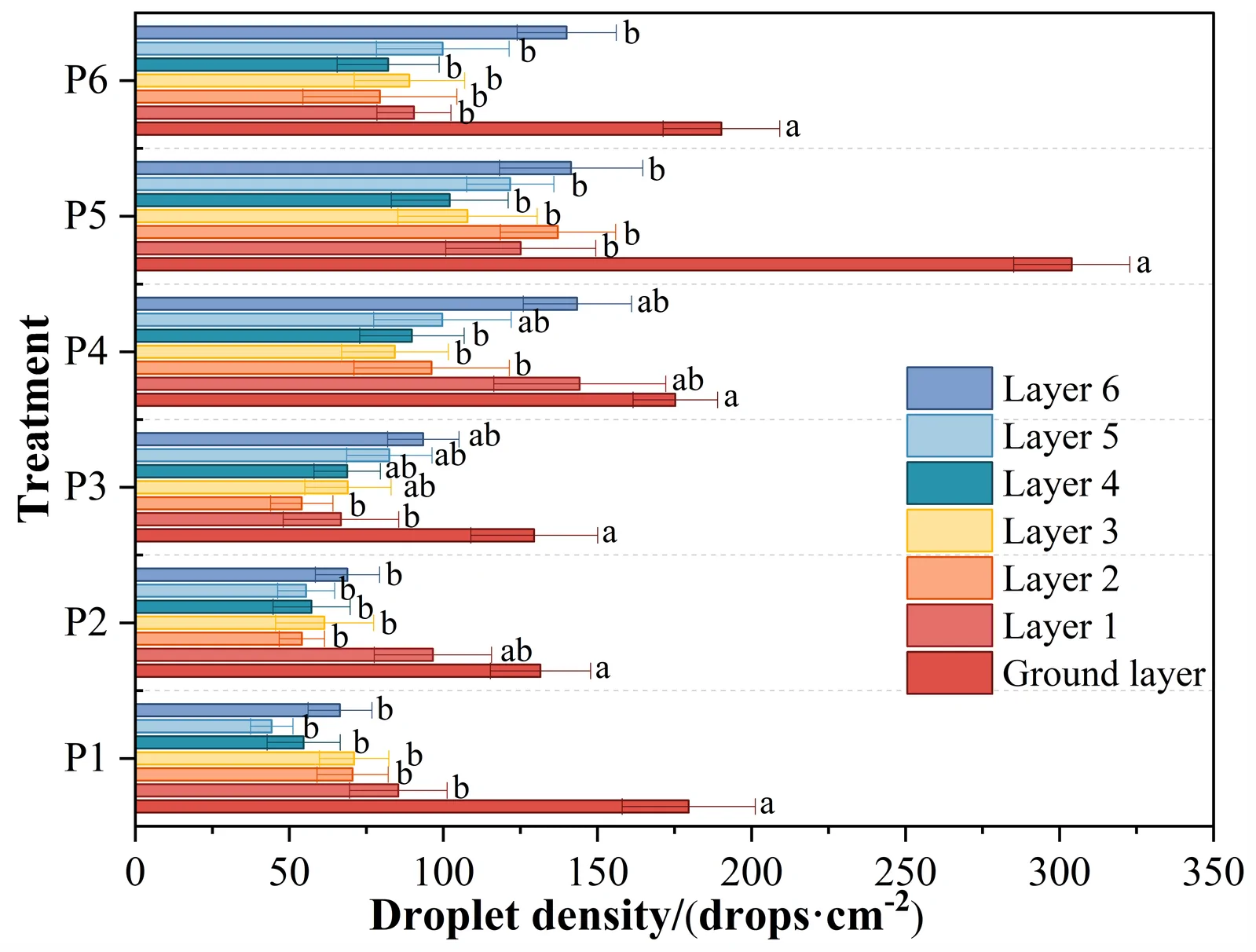
Droplet density across different layers of the vertical canopy profile under different treatments. The same letters indicate no significant differences, whereas different letters indicate significant differences (*p* < 0.05).

The vertical distribution of droplet density for treatments P1–P6 is presented in Figure 4. Overall, the spatial pattern of droplet density was generally consistent with that of droplet coverage. Within the canopy, the highest droplet density was observed in the uppermost canopy layer for most treatments. Across all treatments, the maximum droplet density was 144.3 drops·cm^−2^ (P4, Layer 1), whereas the minimum value was 44.3 drops·cm^−2^ (P1, Layer 5), representing a difference of 100.0 drops·cm^−2^. At the ground sampling positions, the droplet densities were 179.6, 131.5, 129.5, 175.2, 304.0, and 190.2 drops·cm^−2^ for P1–P6, respectively. In all treatments, the ground-layer droplet density exceeded 120.0 drops·cm^−2^ and was generally higher than that measured within the canopy. Among the six treatments, P5 exhibited the highest ground droplet density, further indicating that a greater proportion of droplets penetrated the canopy and were deposited on the ground under the lower flight-speed condition.

The consistently high ground deposition across the treatments also indicates that a considerable proportion of spray droplets was not retained by the target canopy. Therefore, effective UAV spraying should aim to maintain sufficient droplet deposition within the target canopy while reducing droplets passing through the canopy and reaching the ground. This consideration is particularly relevant when optimizing operational parameters, as improvements in canopy penetration should also be evaluated together with spray retention within the target canopy and losses to the ground [45].

#### 2.2.2. Evaluation of Droplet Penetration Within the Vertical Canopy Profile

The mean droplet deposition and corresponding PI for each treatment were calculated to evaluate droplet penetration within the vertical canopy profile. The results are summarized in Table 2. Average droplet coverage ranged from 1.41% to 3.82%, whereas average droplet density ranged from 65.4 to 122.6 drops·cm^−2^ among the treatments. Based on droplet coverage, P1 and P2 exhibited greater overall deposition in the lower canopy than in the upper canopy (PI < 0), whereas the remaining treatments showed the opposite trend (PI > 0), indicating relatively higher deposition in the upper canopy. When evaluated using droplet density, all treatments except P3 exhibited negative PI values, suggesting that droplet density was generally greater in the lower canopy than in the upper canopy.

For the treatments with different atomization levels (P1–P3), increasing the atomization level from L2 to L4 did not produce a consistent trend in canopy penetration, as indicated by the absolute PI values. Nevertheless, a clear difference in deposition tendency was observed. Under the smaller atomization levels (L2 and L3), both droplet coverage and droplet density showed negative PI values, indicating that droplet deposition was generally greater in the lower canopy than in the upper canopy. In contrast, when the atomization level increased to L4, positive PI values were obtained for droplet coverage, indicating that deposition became relatively concentrated in the upper canopy. These results suggest that finer droplets were more likely to penetrate into the lower canopy, which agrees with previous findings reported by Ferguson et al. [46].

The agreement between the present results and previous findings on the penetration of finer droplets indicates that the observed atomization effect is not unique to the present orchard system. However, the present results provide additional context by showing that the benefit of finer droplets should be considered together with the overall deposition pattern. Although finer droplets promoted deposition in lower canopy regions, droplets that penetrate too deeply may also contribute to ground losses when they pass through the canopy without being intercepted [47]. Therefore, the objective of atomization optimization should not simply be to maximize penetration, but to achieve a more balanced vertical distribution while maintaining effective canopy interception.

For the treatments with different flight speeds (P5, P2, and P6), increasing the flight speed from 1.8 to 3.8 m·s^−1^ did not result in a consistent change in the penetration index. This finding suggests that, within the tested range, flight speed had a relatively limited influence on vertical droplet penetration. Nevertheless, additional experiments covering a wider range of operating conditions are required to further clarify the effect of flight speed on canopy penetration.

The absence of a consistent PI response to flight speed also indicates that flight speed alone may not be a sufficiently sensitive indicator of canopy penetration under the tested conditions. Its effect may depend on the simultaneous changes in droplet residence time, rotor-induced airflow, and canopy interception [48]. Therefore, the relatively weak response observed here should not be interpreted as evidence that flight speed is universally unimportant, but rather as a limitation of the tested operating range.

For the treatments with different spray volume rates (P2 and P4), increasing the spray volume rate from 60.0 to 90.0 L·ha^−1^ increased the overall droplet deposition, particularly in the upper canopy. Consequently, the PI based on droplet coverage changed from negative (P2) to positive (P4). However, the increase in spray volume rate did not lead to an obvious improvement in droplet penetration through the canopy. These results indicate that increasing spray volume rate can enhance overall canopy deposition but does not necessarily improve droplet penetration within the vertical canopy profile. It should also be noted that only two spray volume rates were evaluated in this study. Therefore, the observed trends should be interpreted with caution, and additional spray volume rate treatments are needed to establish a more comprehensive understanding of droplet deposition and penetration characteristics.

This result is particularly important when considered together with Experiment I. Experiment I showed that reducing spray volume according to canopy size could avoid excessive deposition on small canopy trees, whereas Experiment II demonstrated that simply increasing spray volume did not necessarily improve vertical penetration. Taken together, the two experiments suggest that UAV spray optimization should not rely on spray volume as the sole control variable. Instead, spray volume should first be adapted to canopy size, while atomization and other operational parameters should subsequently be optimized to regulate the vertical distribution of droplets within the canopy. In this context, the results highlight the need to consider canopy-dependent spray-volume adjustment together with vertical penetration when optimizing UAV orchard spraying.

#### 2.2.3. Frequency Distribution of Droplet Coverage

**Figure 5:**
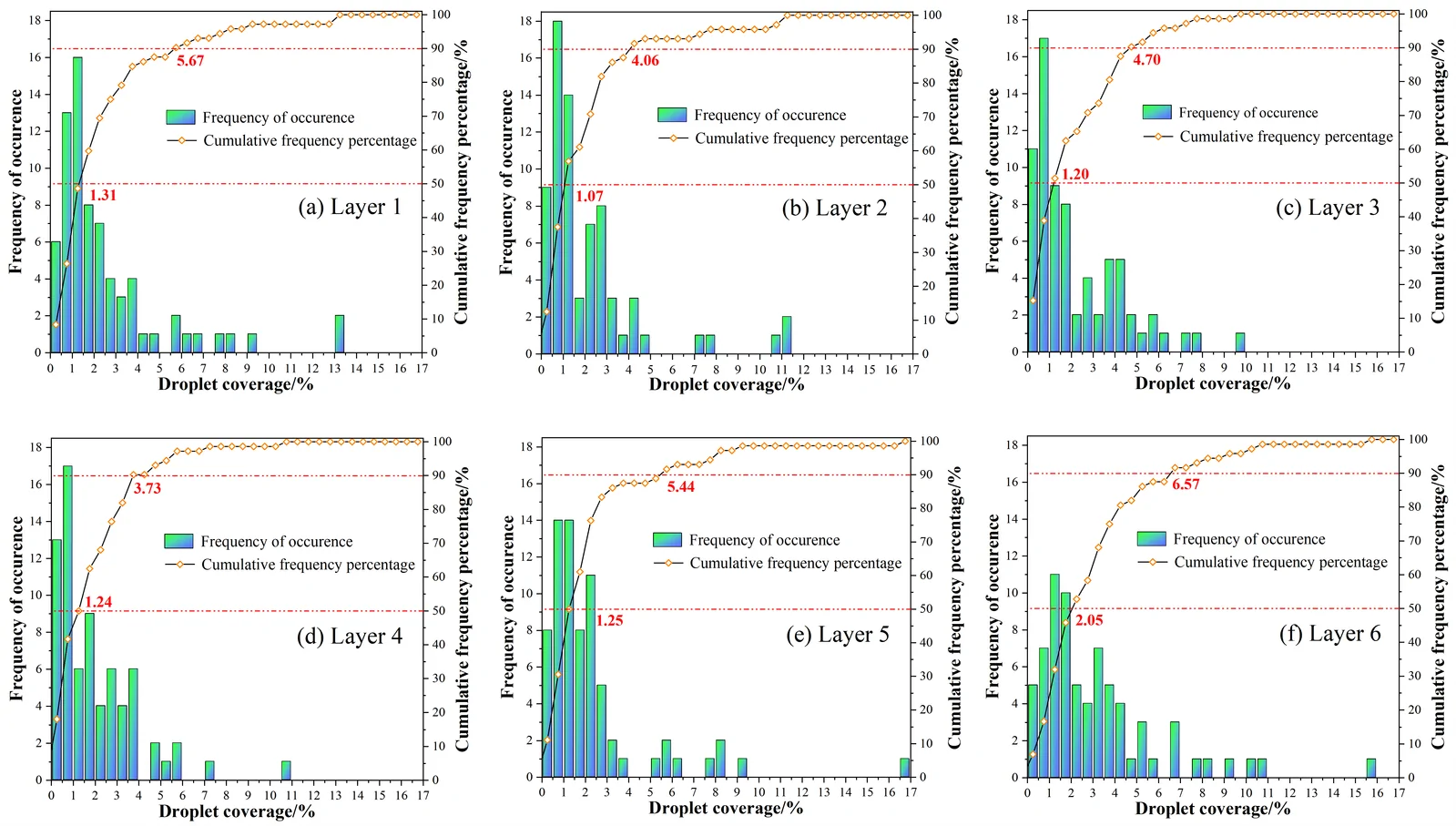
Frequency distribution of droplet coverage across the vertical canopy profile. Red lines indicate the 50% and 90% cumulative frequency levels, and the red values indicate the corresponding droplet coverage.

To further characterize the vertical distribution of droplet deposition within the canopy, the frequency distribution of droplet coverage was analyzed by pooling data from all treatments, irrespective of the operating parameters. The results are presented in Figure 5. Overall, droplet coverage ranged from 0.13% to 16.59%. Accordingly, the histogram was constructed using a maximum coverage value of 17%, a class interval of 0.5%, and 34 classes.

As shown in Figure 5, droplet coverage across the canopy profile was predominantly concentrated within the 0–2% interval. A smaller proportion of observations fell within the 2–5% range, whereas the frequency of droplet coverage decreased markedly when coverage exceeded 5%. The droplet coverage values corresponding to the 50% cumulative frequency were 1.31%, 1.07%, 1.20%, 1.24%, 1.25%, and 2.05% for Layers 1–6, respectively. Except for Layer 6, corresponding to the canopy top, the median coverage values of the remaining canopy layers were remarkably similar, ranging from 1.07% to 1.31%. The relatively higher value observed in Layer 6 further indicates that droplet deposition was concentrated near the canopy top. Similarly, the droplet coverage values corresponding to the 90% cumulative frequency ranged from 3.73% to 6.57%, with the highest value again occurring in Layer 6. These findings provide additional quantitative evidence supporting the vertical deposition pattern described in Section 2.2.1, where the upper canopy generally exhibited greater droplet deposition than the lower canopy.

The frequency distribution analysis further illustrates the differences in vertical deposition among the canopy layers. Layers 1–5 showed relatively similar median coverage values, whereas the canopy-top layer had a higher median coverage, indicating that the uppermost canopy received more spray than the lower canopy layers. At the same time, the wider variation in coverage observed in the lower canopy layers suggests that spray deposition was less consistent at these positions. The frequency distribution therefore provides additional information on the variation in deposition among sampling points within different canopy layers.

#### 2.2.4. Droplet Size Distribution

The percentage distribution of droplets within the seven droplet size classes for all treatments is presented in Table 3. The droplet size distribution was analyzed separately for each sampling layer. Overall, the proportion of droplets decreased progressively with increasing droplet diameter, indicating that most deposited droplets were smaller than 100 μm. Further analysis showed that the proportion of droplets smaller than 100 μm ranged from 64.06% to 74.20% for P1, 67.99% to 74.77% for P2, 56.17% to 66.30% for P3, 54.60% to 60.20% for P4, 63.06% to 71.47% for P5, and 54.89% to 66.26% for P6 across the different sampling layers.

The mean proportion of droplets smaller than 100 μm across all canopy layers was 70.92%, 70.84%, 60.82%, 57.56%, 68.06%, and 62.34% for P1–P6, respectively. For the treatments with different atomization levels (P1–P3), the proportion of droplets smaller than 100 μm gradually decreased as the atomization level increased from L2 to L4, which was consistent with the expected increase in droplet size associated with coarser atomization settings. For the treatments with different flight speeds (P5, P2, and P6), the proportion of droplets smaller than 100 μm first increased and then decreased as flight speed increased from 1.8 to 3.8 m·s^−1^. This result suggests that both excessively low and excessively high flight speeds may influence the deposited droplet size distribution, while the underlying mechanism by which flight speed influences droplet size characteristics requires further investigation. For the treatments with different spray volume rates (P2 and P4), increasing the spray volume rate from 60.0 to 90.0 L·ha^−1^ substantially reduced the proportion of droplets smaller than 100 μm, with a difference of 13.28% points between the two treatments. Moreover, P4 exhibited the lowest mean proportion of droplets smaller than 100 μm among all six treatments. These results indicate that increasing the spray volume rate during UAV orchard spraying shifted the deposited droplet population toward larger droplet sizes.

The droplet size results also help explain the penetration patterns observed in the PI analysis. The higher proportion of small droplets under the finer atomization treatments was associated with greater deposition in the lower canopy, whereas coarser atomization shifted deposition toward the upper canopy. These results provide further information on the role of droplet size in determining vertical deposition under UAV spraying. The three operational parameters examined in Experiment II showed different effects on the deposited droplet-size distribution. Atomization level produced a clear trend, with the proportion of droplets smaller than 100 μm decreasing as atomization became coarser. Flight speed showed a non-monotonic effect, with the proportion of droplets smaller than 100 μm first increasing and then decreasing as flight speed increased. Increasing spray volume from 60.0 to 90.0 L·ha^−1^ also reduced the proportion of droplets smaller than 100 μm, indicating a shift in the deposited droplet population toward larger sizes. These results suggest that spray volume, atomization level, and flight speed should all be considered when selecting operational parameters for UAV orchard spraying, as each parameter may affect the characteristics and vertical distribution of deposited droplets in a different manner.

Several limitations should nevertheless be considered when interpreting these droplet-size results. First, the tested spray-volume treatments were limited to 60.0 and 90.0 L·ha^−1^, which is insufficient to establish a continuous relationship between spray volume and deposited droplet-size distribution. Second, the experiments were conducted under the specific orchard, meteorological, and operational conditions described in this study. The observed relationships may therefore change under different canopy structures, wind conditions, flight heights, or UAV configurations. In particular, the non-monotonic response to flight speed suggests that the effect of flight speed on deposited droplet-size distribution may involve multiple interacting factors and requires further investigation [8,29,49]. Further experiments covering a broader range of canopy and operational conditions are needed to establish more generalizable relationships among spray volume, atomization level, flight speed, droplet size, and canopy penetration.

## 3. Materials and Methods

### 3.1. Experimental Site

The experiments were conducted in a commercial Nanguo pear orchard located in Jiewen Town, Haicheng City, Liaoning Province, China (123°3′14″ E, 40°46′20″ N). The orchard was situated on a mountainous slope with an average gradient of approximately 40°, and Nanguo pear trees were selected as the spray target, with a planting density of approximately 525 trees·ha^−1^. To systematically investigate the canopy size effect on UAV spraying in mountainous orchards, two experiments were conducted. Experiment I focused on canopy size-based spray volume optimization and droplet deposition evaluation, whereas Experiment II investigated droplet deposition and penetration characteristics along the vertical canopy profile.

### 3.2. UAV Model and Spraying Parameters

**Figure 6:**
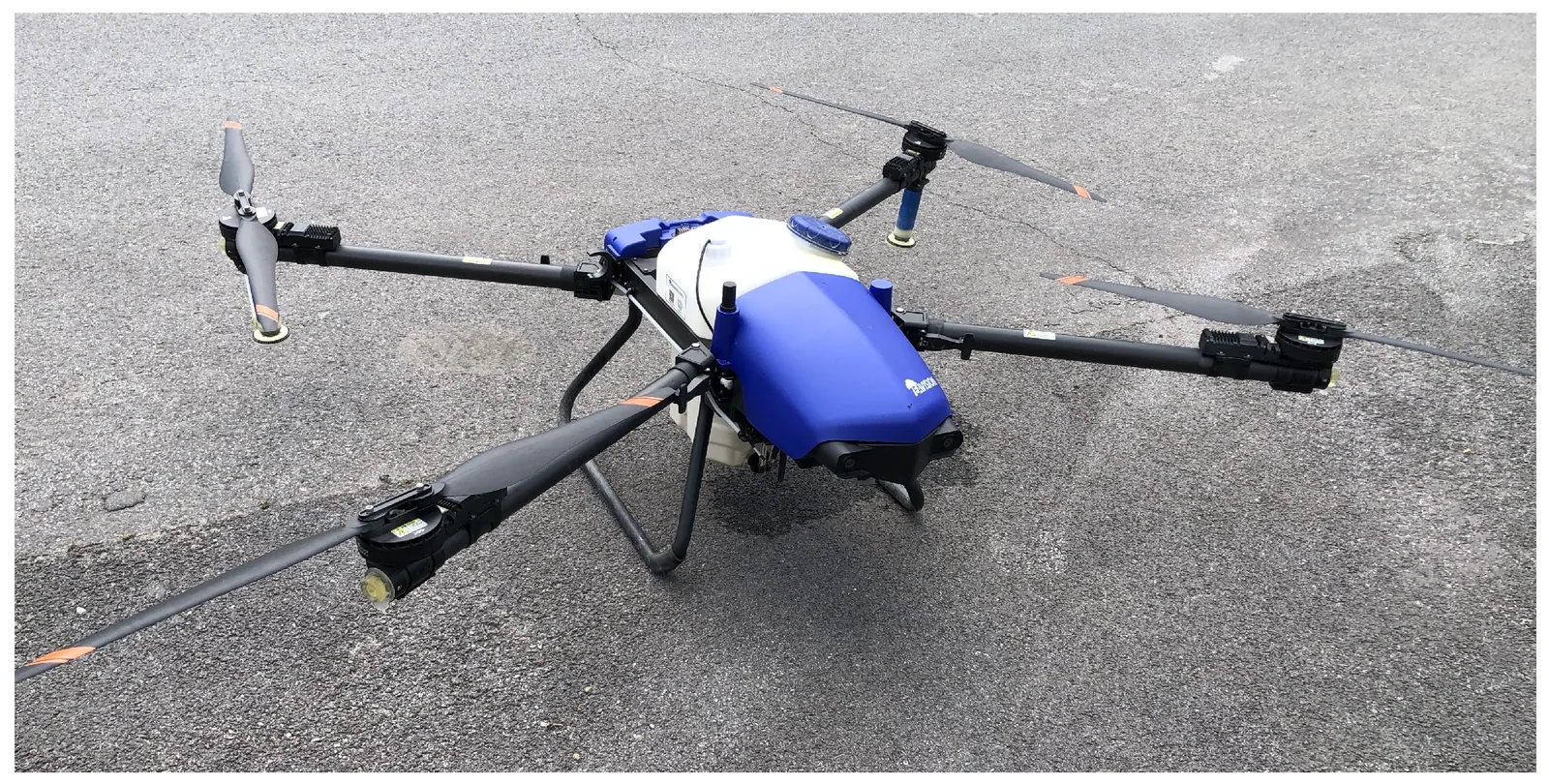
EA-30X UAV used in the spraying experiments.

All spraying operations were performed using an EA-30X plant protection UAV (Suzhou EAVISION Robotic Technologies Co., Ltd., Suzhou, China) (Figure 6). The UAV was equipped with CCMS-L20000 twin-peak atomizing nozzles, whose atomization could be adjusted through five atomization levels (L1–L5) by changing the nozzle operating mode [39]. According to the manufacturer’s technical specifications, the five atomization levels (L1–L5) correspond to nominal droplet sizes of 20, 30, 40, 100, and 250 μm, respectively. Throughout all experiments, the flight height was maintained at 2.5 m above the canopy top.

The main operating parameters investigated in this study included spray volume rate, flight speed, and atomization level. Spray volume rates of 30.6, 34.2, 60.0, and 90.0 L·ha^−1^, flight speeds of 1.8, 2.8, and 3.8 m·s^−1^, and atomization levels of L2, L3, and L4 were evaluated. Different combinations of these parameters were assigned to Experiments I and II. For each treatment, the target spray volume rate was preset in the UAV ground control system together with the specified flight speed and flight path parameters. During operation, the spraying system automatically regulated the spray flow rate according to the preset operational parameters to achieve the target spray volume rate. Water was used instead of pesticide solution in all spraying trials to eliminate the influence of spray formulation properties on droplet deposition measurements.

### 3.3. Experiment I: Canopy Size-Based Spray Volume Optimization and Droplet Deposition Evaluation

Experiment I was designed to compare droplet deposition between trees with different canopy sizes under both a constant spray volume rate and canopy-adjusted spray volume rates. According to canopy dimensions, two representative tree types were selected and designated as canopy size A (large canopy) and canopy size B (small canopy). The measured canopy parameters included row spacing, tree spacing, tree height, canopy thickness, canopy width along the row direction, canopy width perpendicular to the row direction, LWA and TRV.

**Figure 7:**
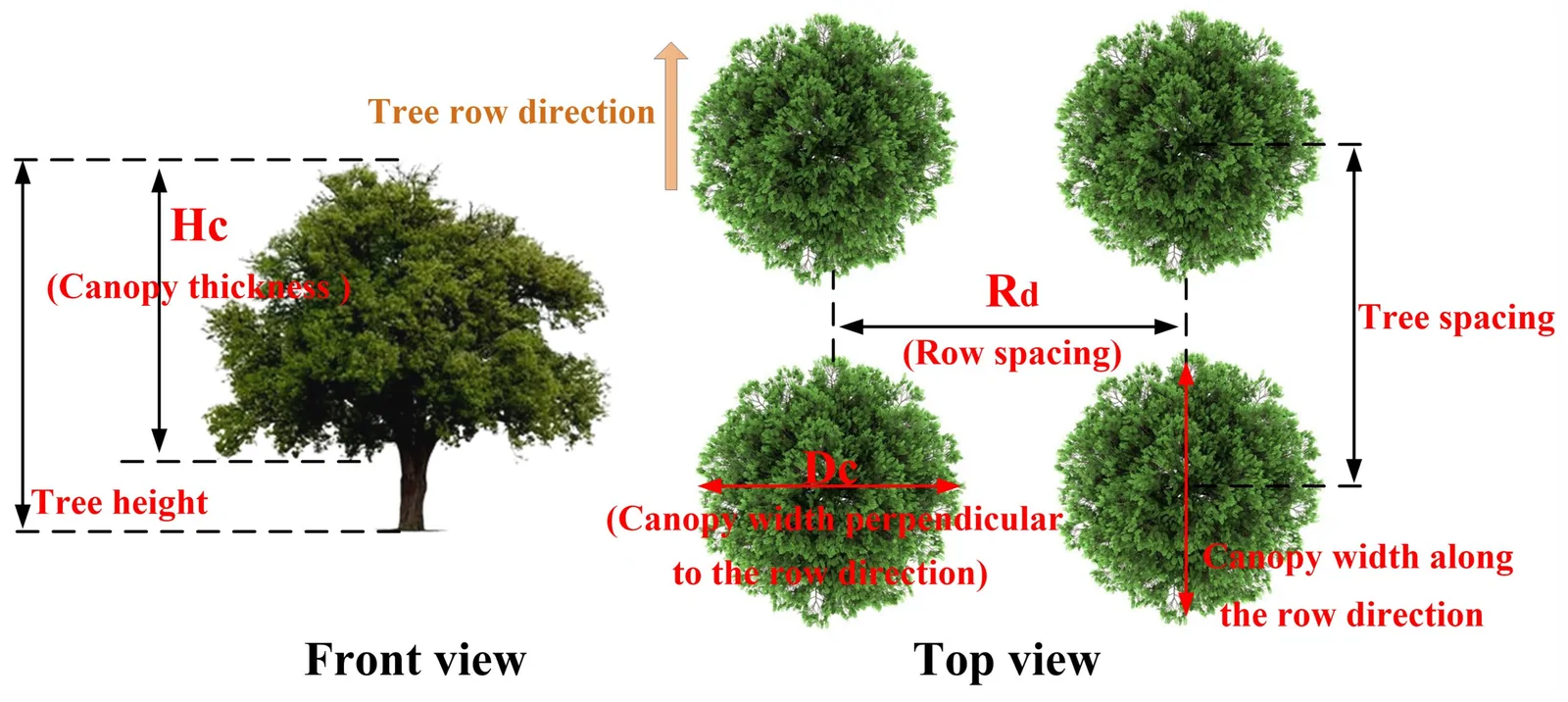
Schematic diagram of the characteristic parameters of the Nanguo pear tree.

The LWA (m^2^·ha^−1^) and TRV (m^3^·ha^−1^) are calculated according to the formulas [16,50,51]:

(1) $$\mathrm{LWA}=\frac{2\times H_{C}\times 10000}{R_{d}},$$

(2) $$\mathrm{TRV}=\frac{D_{C}\times H_{C}\times 10000}{R_{d}},$$

where *H_C_* is the canopy thickness (m), *D_C_* is the canopy width perpendicular to the row direction used for TRV calculation (m), and Rd is the row spacing (m). LWA and TRV are established canopy characterization indices used to describe the spatial scale of tree canopies. LWA characterizes the leaf wall area associated with a tree row, whereas TRV represents the three-dimensional volume occupied by the tree canopy. Although their calculation is primarily based on geometric canopy parameters, these indices provide a standardized basis for relating canopy size to spray volume. In this study, LWA and TRV were used as canopy-size indicators to adjust the spray volume according to differences in canopy size. A schematic illustration of the canopy parameters is shown in Figure 7. Detailed characteristics of the two canopy types are presented in Table 4.

In experiment I, four spray treatments (T1–T4) were established. For all treatments, the flight height was maintained at 2.5 m above the canopy, and the flight speed was fixed at 2.8 m·s^−1^. Each treatment was replicated three times. For every replicate, new water-sensitive papers (WSP, 26 mm × 76 mm, Syngenta Crop Protection AG, Basel, Switzerland) were deployed, and an independent UAV spraying operation was conducted. The same group of trees was used for all treatments to ensure consistent sampling. As water was used as the spray medium, each spray application was performed only after the previous spray had completely dried. T1 used canopy size A with a spray volume rate of 60.0 L·ha^−1^ and served as the conventional reference treatment. T2 applied the same spray volume rate to canopy size B to evaluate the influence of canopy size under a constant application rate. T3 and T4 also targeted canopy size B, but the spray volume rates were determined using the LWA and TRV methods, respectively. The spray volume rates based on the LWA and TRV methods are calculated as follows:

(3) $$A_{\mathrm{LWA}}=\mathrm{LWA}\times I_{\mathrm{LWA}},$$

(4) $$A_{\mathrm{TRV}}=\mathrm{TRV}\times I_{\mathrm{TRV}},$$

where I_LWA_ (L·m^−2^) and I_TRV_ (L·m^−3^) represent the application coefficients for the LWA and TRV methods, respectively. These coefficients represent the spray volume required per unit LWA or TRV and establish the quantitative relationship between canopy size and spray volume.

For the reference treatment T1, the LWA and TRV of canopy size A were 17,500 m^2^·ha^−1^ and 24,500 m^3^·ha^−1^, respectively, and the spray volume rate was 60.0 L·ha^−1^. Accordingly, the application coefficients were calculated as I_LWA_ = 0.00342 L·m^−2^ and I_TRV_ = 0.00245 L·m^−3^. These coefficients were then applied to the LWA and TRV values of canopy size B (10,000 m^2^·ha^−1^ and 12,500 m^3^·ha^−1^) to determine the adjusted spray volume rates for T3 and T4, respectively. Thus, the spray volume rate for T3 is calculated as 10,000 × 0.00342 = 34.2 L·ha^−1^, while that for T4 is calculated as 12,500 × 0.00245 = 30.6 L·ha^−1^. The spray parameters for each treatment are summarized in Table 5.

### 3.4. Experiment II: Droplet Deposition and Penetration Characteristics Along the Vertical Canopy Profile

Experiment II was conducted to investigate the effects of atomization level, spray volume rate, and flight speed on droplet deposition and penetration within the vertical canopy profile of Nanguo pear trees. The treatment combinations are summarized in Table 6. A total of six treatments (P1–P6) were established following the principle of controlling a single variable. P1, P2, and P3 were designed to evaluate the effect of atomization level on vertical droplet penetration, using atomization levels L2–L4 (30, 40, and 100 μm nominal droplet sizes, respectively). P2 and P4 were compared to assess the effect of spray volume rate. The spray volume rates of 60 and 90 L·ha^−1^ were selected based on the practical UAV spraying conditions of the orchard and our previous field experience with mountainous Nanguo pear orchards [7,8,22,28,29,39]. P5, P2, and P6 were used to investigate the effect of flight speed while keeping the other operational parameters constant, with flight speeds of 1.8, 2.8, and 3.8 m·s^−1^, respectively. Each treatment was replicated three times. For every replicate, new WSPs were deployed, and an independent UAV spraying operation was conducted. The same group of trees was used for all treatments to ensure consistent sampling. As water was used as the spray medium, each spray application was performed only after the previous spray had completely dried.

### 3.5. Sampling Scheme and Droplet Collection

**Figure 8:**
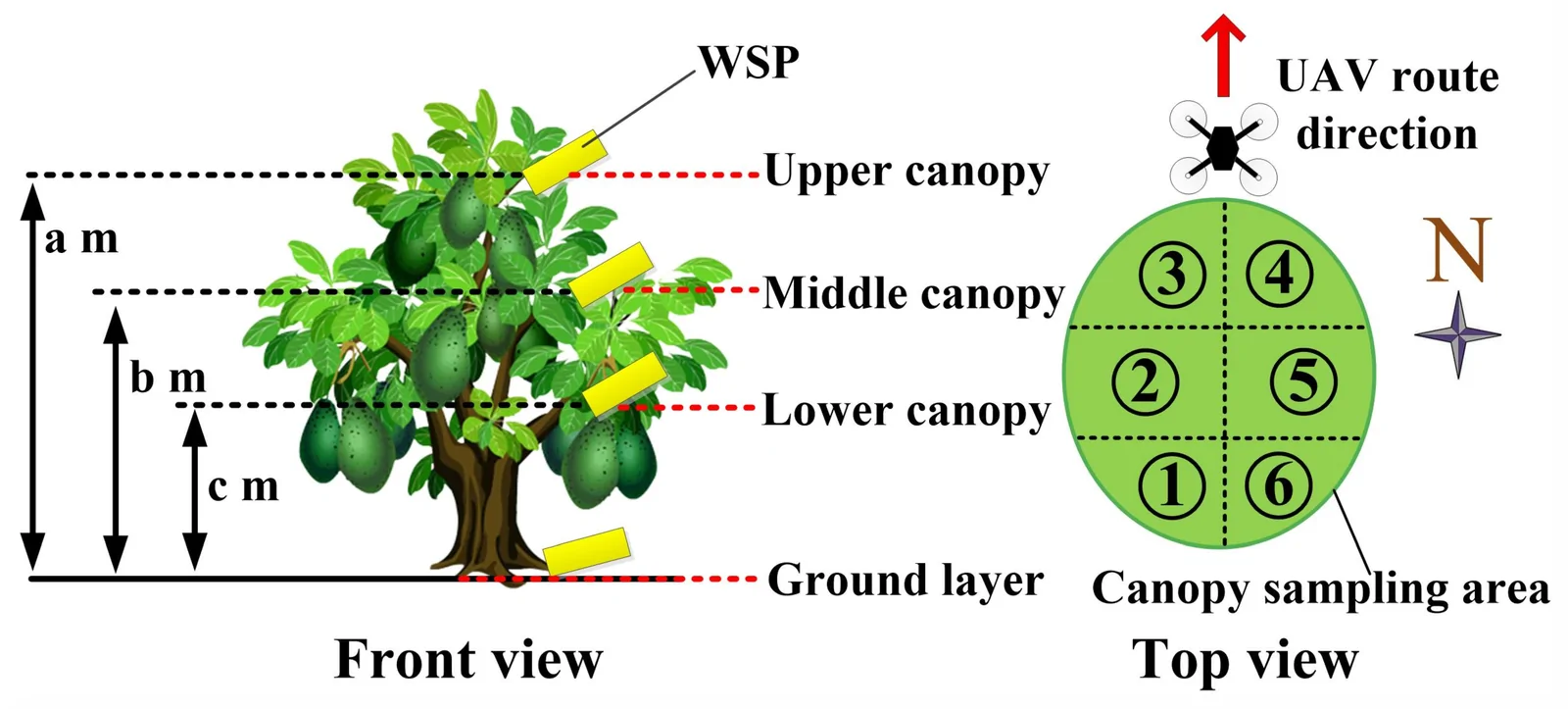
Arrangement of sampling points in the tree canopy and on the ground.

A conventional three-layer canopy sampling strategy was adopted in Experiment I, as illustrated in Figure 8. The canopy was divided into upper, middle, and lower layers. For canopy size A, the sampling heights of the upper (a), middle (b), and lower (c) layers were 2.9, 1.9, and 1.0 m, respectively, whereas those for canopy size B were 2.0, 1.5, and 1.0 m, respectively. Each canopy layer was further divided into six sampling zones. One WSP was attached to the adaxial surface and another WSP was attached to the abaxial surface of leaves in each sampling zone to quantify droplet deposition throughout the canopy. In addition, six WSPs were placed on the ground directly beneath each tree canopy to evaluate the droplet deposition that reached the ground after penetrating the canopy. The three-layer sampling design was selected according to the objective of Experiment I, which was to compare the overall droplet deposition characteristics between trees with different canopy sizes under different spray volume treatments. The upper, middle, and lower layers were used to represent the major vertical regions of the canopy, providing sufficient vertical information to characterize the overall deposition pattern while maintaining practical feasibility for repeated field sampling.

**Figure 9:**
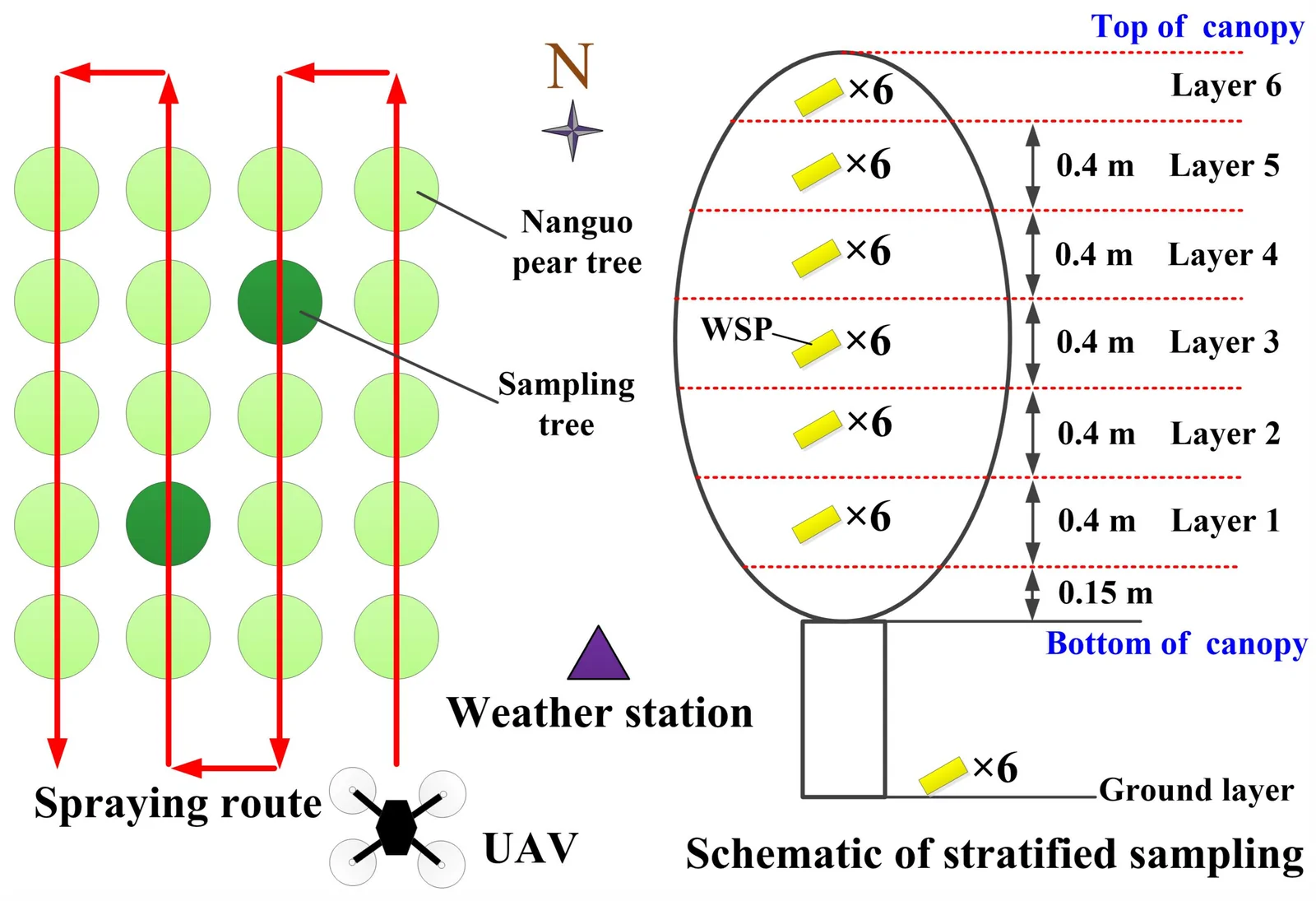
Schematic diagram of UAV spraying operation and canopy sampling scheme.

The sampling layout for Experiment II is shown in Figure 9. Two Nanguo pear trees with similar growth characteristics were randomly selected as representative sample trees. Starting 0.15 m above the bottom of the canopy, sampling layers were established at 0.4 m vertical intervals, resulting in six canopy layers (Layers 1–6) from bottom to top. For each sample tree, six WSPs were randomly distributed within each canopy layer, giving a total of 36 WSPs per tree and 72 WSPs for the two sample trees. In addition, six WSPs were deployed on the ground beneath each tree, resulting in 12 ground sampling points for evaluating droplet deposition after canopy penetration and the associated potential ground loss. In contrast to Experiment I, Experiment II was specifically designed to investigate the vertical penetration and distribution of droplets within the canopy. Therefore, a six-layer sampling scheme was adopted to provide a higher vertical spatial resolution. The 0.4 m vertical interval allowed the variation in droplet deposition to be characterized in greater detail from the lower to the upper canopy, thereby facilitating the evaluation of vertical deposition gradients and canopy penetration under different spraying treatments.

**Figure 10:**
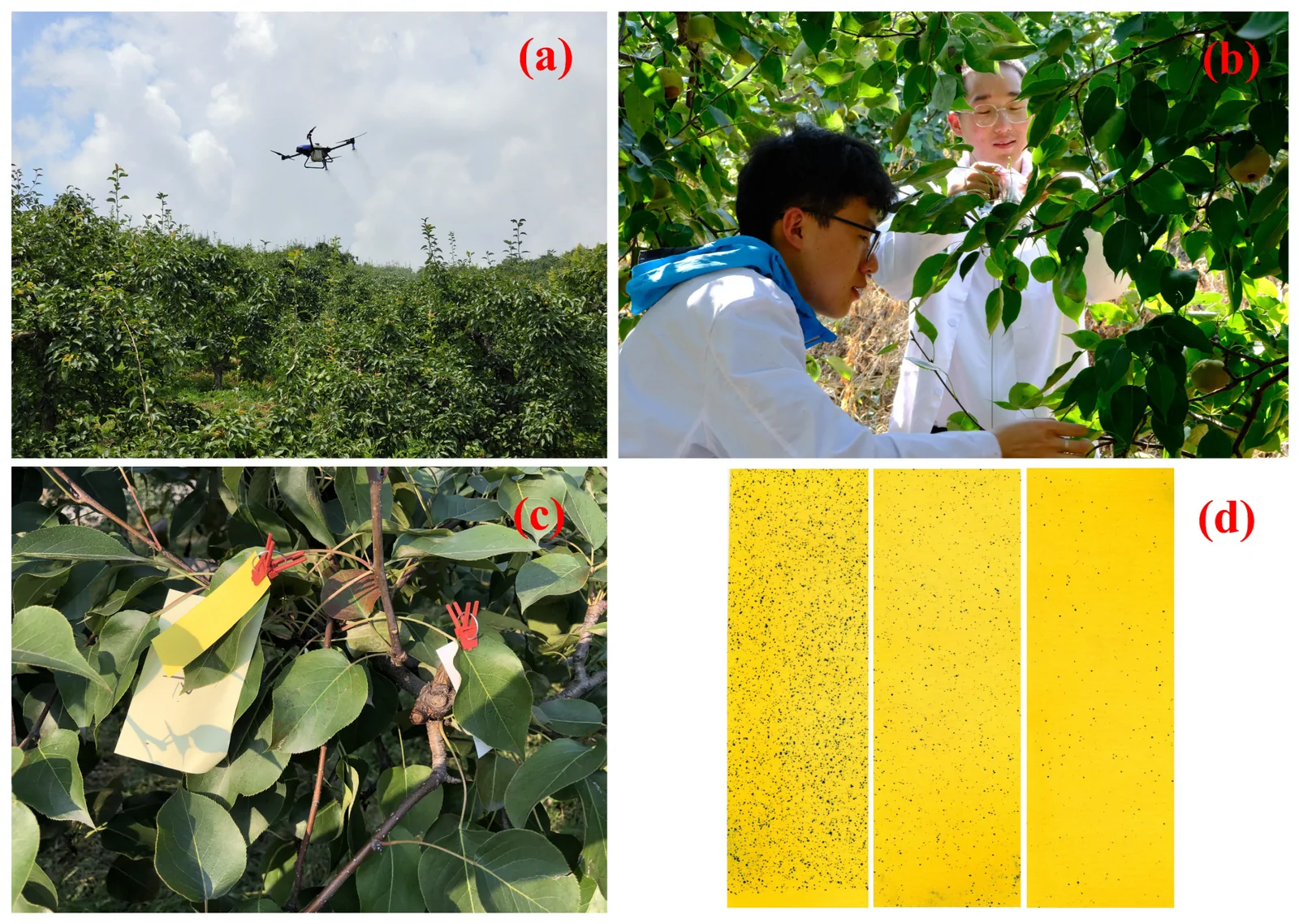
Photographs of the UAV spraying and sampling operations in the mountainous Nanguo pear orchard. (**a**) UAV spraying operation in the orchard; (**b**) placement of WSPs for droplet sampling; (**c**) deployed WSPs during field sampling; and (**d**) scanned images of WSPs after sampling.

Figure 10 shows photographs of the actual field experimental conditions, UAV spraying operation, and on-site WSP sampling procedures in the mountainous Nanguo pear orchard. These photographs provide a direct representation of the field environment and experimental procedures.

During spraying, the UAV flew along the tree rows in a back-and-forth flight pattern. After each UAV spraying operation, the WSPs were collected sequentially by the operators wearing gloves after the surfaces had dried [44]. The collected WSPs were placed into individual sealed bags and stored in a cool, dark environment to minimize the influence of external conditions on the results.

### 3.6. Meteorological Measurements

Meteorological conditions were monitored throughout the experiments using a Kestrel 5500 Link portable weather station (Nielsen-Kellerman, Boothwyn, PA, USA), which was installed approximately 3 m above the ground and positioned away from the UAV flight path to avoid interference from rotor airflow. Air temperature, relative humidity, wind speed, and wind direction were recorded at 2 s intervals. The meteorological data were used to verify that environmental conditions satisfied the operational requirements for UAV spraying and to provide reference information for interpreting droplet drift, canopy deposition, and ground deposition [42,49]. During Experiment I, the average wind speed was 0.6 ± 0.2 m·s^−1^, with a prevailing southerly wind, an average air temperature of 33.4 ± 0.6 °C, and a relative humidity of 58.8 ± 1.8%. During Experiment II, the corresponding values were 0.8 ± 0.4 m·s^−1^, a prevailing southerly wind, 31.7 ± 0.7 °C, and 54.6 ± 1.5%, respectively.

### 3.7. Droplet Analysis

After collection, all WSPs were scanned individually using a flatbed scanner at a resolution of 600 dpi. The scanned images were analyzed using DepositScan (USDA-ARS Application Technology Research Unit, Wooster, OH, USA) to quantify droplet deposition parameters, including the volume median diameter (D_V0.5_), droplet coverage, and droplet density [52]. To further characterize the vertical distribution of droplet deposition within the canopy, the frequency distributions of droplet coverage were summarized for each sampling layer. Based on these distributions, the droplet coverage values corresponding to 50% and 90% cumulative frequencies were determined to provide a more comprehensive evaluation of deposition characteristics throughout the canopy profile.

Furthermore, to better characterize droplet deposition within the canopy and on the ground, droplets were classified into different size classes according to the diameters. Considering the atomization characteristics of the EA-30X UAV, droplet diameters were grouped into seven classes: <75 μm, 76–100 μm, 101–125 μm, 126–150 μm, 151–175 μm, 176–200 μm, and >200 μm. The percentage of droplets within each size class was then calculated for each treatment.

### 3.8. Penetration Index

The penetration index (PI) was proposed to quantitatively evaluate droplet penetration within the vertical canopy profile, particularly for the multi-layer sampling strategy adopted in this study. The PI is calculated as follows:

(5) $$\mathrm{PI}=\sum_{i=2}^{n}\left(\sum_{j=1}^{i-1}\left(\frac{D_{i}}{D_{j}}-1\right)\right),$$

where PI represents the penetration index calculated from either droplet coverage or droplet density and *n* is the number of canopy sampling layers (*n* = 6 in this study). When PI is calculated based on droplet coverage, *D_i_* and *D_j_* represent the droplet coverage of the *i*-th and *j*-th canopy layers, respectively; when PI is calculated based on droplet density, *D_i_* and *D_j_* represent the droplet density of the *i*-th and *j*-th canopy layers, respectively, with *i* > *j*.

The PI was calculated by sequentially comparing the same deposition metric between each canopy layer and all lower layers from bottom to top, and summing the resulting relative differences. Specifically, the canopy was divided into multiple vertical sampling layers and numbered sequentially from the bottom upward. For each layer *i*, its droplet deposition *D_i_* was compared with the deposition *D_j_* of each lower layer *j* (*j* = 1, …, *i* − 1) by calculating the relative difference (*D_i_*/*D_j_* − 1). The relative differences from all layer-to-layer comparisons were then summed to obtain the PI. A PI value closer to zero indicates a more uniform vertical distribution of droplets throughout the canopy. Under Equation (5), PI can take either negative or positive values depending on the relative droplet deposition between different canopy layers. A negative PI indicates that droplet deposition was generally greater in the lower canopy than in the upper canopy, whereas a positive PI indicates greater deposition in the upper canopy.

### 3.9. Statistical Analysis

Experimental data were organized and preliminarily processed using Microsoft Excel 2019 (Microsoft Corporation, Redmond, WA, USA). Figures were prepared using Origin 2021 (OriginLab Corporation, Northampton, MA, USA), and statistical analyses were performed using SPSS Statistics 25 (IBM, Armonk, NY, USA). Data involving multiple groups were analyzed using one-way analysis of variance (ANOVA), followed by Fisher’s least significant difference (LSD) post hoc test. Comparisons between two groups were performed using an independent-samples *t*-test. When the assumptions of parametric tests were not met, appropriate non-parametric tests were applied. Statistical significance was set at *p* < 0.05.

## 4. Conclusions

This study evaluated UAV-based precision spraying in Nanguo pear orchards by investigating the effects of canopy size and operational parameters on droplet deposition and vertical canopy penetration. The main conclusions are as follows:

(1) Under the conditions investigated, canopy size was associated with differences in droplet deposition during UAV orchard spraying. Under a constant spray volume rate of 60.0 L·ha^−1^, small canopy trees, owing to their relatively sparse canopy structure, exhibited substantially greater droplet deposition in the upper and middle canopy layers than large canopy trees. Meanwhile, significantly higher droplet coverage and density were detected on the ground beneath the canopy, indicating excessive deposition and increased spray losses. These results demonstrate that applying a uniform spray volume regardless of canopy size is unsuitable for precision orchard spraying.
(2) LWA- and TRV-based spray volume adjustment reduced the spray volume rate for small canopy trees while maintaining comparable deposition in the upper and middle canopy layers. The spray volume rate was reduced from 60.0 L·ha^−1^ to 34.2 L·ha^−1^ and 30.6 L·ha^−1^, respectively, while achieving deposition levels in the upper and middle canopy comparable to those of large canopy trees. These findings demonstrate the feasibility of canopy size-based spray volume adjustment for reducing spray use without compromising deposition performance. However, both methods provided limited improvement in droplet deposition within the lower canopy and on abaxial leaf surfaces, indicating that conventional LWA- and TRV-based approaches developed for ground sprayers cannot fully accommodate the top-down deposition characteristics of UAV spraying.
(3) Under the tested operating conditions, droplet penetration within the vertical canopy profile was influenced by atomization level, spray volume rate, and flight speed, with atomization level showing the clearest association with the vertical deposition pattern. Droplet coverage was predominantly distributed within the 0–2% range, while deposition was generally greatest at the canopy top. Droplet size distribution analysis showed that 57.56–70.92% of deposited droplets were smaller than 100 μm. Compared with the coarser L4 atomization level, the finer L2 and L3 atomization levels promoted droplet penetration into the lower canopy, whereas larger droplets were more likely to be intercepted by the upper canopy.
(4) Spray volume rate and flight speed showed different effects on droplet deposition and should therefore be considered together with atomization level when optimizing UAV spraying parameters. Within the tested range (1.8–3.8 m·s^−1^), flight speed did not produce a consistent monotonic effect on the PI. Increasing the spray volume rate from 60.0 to 90.0 L·ha^−1^ increased overall droplet deposition and the size of deposited droplets but did not result in a corresponding improvement in vertical canopy penetration.

Overall, the results of this study suggest that canopy size, spray volume rate, atomization level, and flight parameters should be considered when optimizing UAV spraying in mountainous orchards to balance effective target deposition, canopy penetration, and reduced ground deposition and potential spray losses. However, the findings are subject to the specific experimental conditions and should be regarded as a reference for similar conditions rather than as broadly applicable recommendations for UAV spraying. Future research should further integrate three-dimensional canopy structure, rotor-induced airflow, and droplet transport characteristics, together with broader field validation, to develop variable-rate spraying models with improved applicability across different UAV orchard spraying conditions. This work could provide a theoretical and technical basis for precision variable-rate spraying with UAVs.

## Figures and Tables

**Table 1:** Droplet deposition parameters on abaxial leaf surfaces at different sampling layers.

Sampling Layer	Parameters	T1	T2	T3	T4
Upper layer	Droplet coverage (%)	0.96	1.09	2.98	0.73
	Droplet density (drops/cm^2^)	48.6	58.3	138.8	34.4
	D_V0.5_ (μm)	119	114	125	122
Middle layer	Droplet coverage (%)	1.01	0.11	0.51	0.25
	Droplet density (drops/cm^2^)	53.9	4.3	29.1	14.7
	D_V0.5_ (μm)	119	144	113	104
Lower layer	Droplet coverage (%)	5.13	0.63	1.25	0.21
	Droplet density (drops/cm^2^)	227.1	34.4	65.8	11.5
	D_V0.5_ (μm)	143	126	115	119

**Table 2:** Summary of droplet deposition values and PI within the vertical canopy profile for each treatment.

Treatments	Droplet Coverage (%)		Droplet Density (Drops/cm^2^)	
	Mean ± SD	PI	Mean ± SD	PI
P1	1.42 ± 0.35	−0.14	65.4 ± 14.3	−0.32
P2	1.41 ± 0.32	−0.04	65.6 ± 16.1	−0.21
P3	2.09 ± 0.53	0.37	72.4 ± 13.7	0.03
P4	3.82 ± 1.11	0.17	109.6 ± 27.0	−0.14
P5	3.00 ± 0.66	0.15	122.6 ± 15.6	−0.19
P6	2.69 ± 0.59	0.25	96.8 ± 22.4	−0.03

**Table 3:** Percentage (%) of droplets in each droplet size range across sampling layers for each treatment.

Treatments	Droplet Size Range/μm	Sampling Layer						
		Ground Layer	Layer 1	Layer 2	Layer 3	Layer 4	Layer 5	Layer 6
P1	<75	42.53	43.51	40.87	35.68	43.33	43.45	43.05
	76~100	29.11	30.69	29.60	28.38	28.62	28.51	29.12
	101~125	14.53	14.12	13.17	16.12	15.26	14.07	14.05
	126~150	7.87	7.22	9.63	11.37	7.59	8.11	8.80
	151~175	3.41	2.79	4.05	4.86	3.22	3.64	3.48
	176~200	1.29	1.09	1.32	2.20	1.21	1.33	0.85
	>200	1.25	0.59	1.38	1.40	0.76	0.89	0.66
P2	<75	40.96	44.74	42.75	39.02	43.72	37.45	38.37
	76~100	30.78	30.03	28.73	28.97	27.34	32.51	30.53
	101~125	14.71	13.37	15.47	15.40	15.03	15.88	15.64
	126~150	8.26	6.99	8.29	9.74	8.19	8.10	9.16
	151~175	3.20	2.87	3.06	4.40	3.49	3.67	4.25
	176~200	1.31	1.15	0.98	1.20	1.37	1.42	1.49
	>200	0.79	0.86	0.72	1.26	0.85	0.97	0.55
P3	<75	33.42	37.65	35.18	34.91	33.73	30.50	31.65
	76~100	27.48	28.65	28.76	26.32	26.22	26.75	24.52
	101~125	14.99	15.78	15.45	16.67	16.93	16.61	17.16
	126~150	10.79	10.53	11.36	11.14	11.30	12.97	13.78
	151~175	6.14	4.21	5.85	5.10	6.13	6.50	6.45
	176~200	3.47	1.39	2.09	2.67	2.99	3.64	3.04
	>200	3.71	1.79	1.31	3.19	2.71	3.03	3.40
P4	<75	32.40	33.12	32.88	32.06	30.59	29.93	29.32
	76~100	27.05	27.08	27.26	24.78	26.41	24.75	25.28
	101~125	15.07	16.36	17.09	17.52	17.53	16.62	16.90
	126~150	11.14	11.32	11.51	13.07	12.40	13.28	12.19
	151~175	5.94	5.45	5.67	6.11	6.49	7.04	7.15
	176~200	3.56	2.97	2.59	2.68	2.89	3.30	3.82
	>200	4.83	3.71	3.00	3.78	3.68	5.08	5.33
P5	<75	36.77	40.74	35.27	40.03	42.23	38.25	38.28
	76~100	26.29	30.72	29.53	30.32	29.24	29.08	29.65
	101~125	15.59	14.38	16.21	14.25	15.02	15.71	15.70
	126~150	10.15	9.08	10.70	8.73	8.22	9.32	9.14
	151~175	5.17	3.08	4.82	3.44	3.49	4.13	4.18
	176~200	2.65	1.24	2.01	1.76	1.11	1.82	1.72
	>200	3.38	0.74	1.46	1.47	0.70	1.68	1.33
P6	<75	37.74	35.72	35.19	26.95	30.99	33.77	33.76
	76~100	28.52	30.20	29.94	27.94	27.84	29.81	28.01
	101~125	15.50	16.95	17.26	17.66	16.67	17.12	17.26
	126~150	8.98	10.05	9.92	14.11	11.82	11.28	11.56
	151~175	4.93	4.11	4.94	7.39	6.16	4.67	5.31
	176~200	2.31	1.59	1.47	3.29	2.94	2.21	2.10
	>200	2.02	1.38	1.28	2.66	3.59	1.15	2.00

**Table 4:** Characteristic parameters of Nanguo Pear trees with different canopy sizes.

Parameters	Canopy Size A	Canopy Size B
Row spacing (m)	4	4
Tree spacing (m)	3.5	3.5
Tree height (m)	4	2.5
Canopy thickness (m)	3.5	2
Canopy width along the row direction (m)	2.6	2.1
Canopy width perpendicular to the row direction (m)	2.8	2.5
LWA (m^2^/ha)	17,500	10,000
TRV (m^3^/ha)	24,500	12,500

**Table 5:** Parameter settings of spray treatments in Experiment I.

Treatments	Canopy Size	Flight Height (m)	Flight Speed (m/s)	Spray Volume Rate (L/ha)
T1	A	2.5	2.8	60.0
T2	B	2.5	2.8	60.0
T3	B	2.5	2.8	34.2
T4	B	2.5	2.8	30.6

**Table 6:** Parameter settings of spray treatments in Experiment II.

Treatments	Atomization Level	Spray Volume Rate (L/ha)	Flight Height (m)	Flight Speed (m/s)
P1	L2 (30 μm)	60.0	2.5	2.8
P2	L3 (40 μm)	60.0	2.5	2.8
P3	L4 (100 μm)	60.0	2.5	2.8
P4	L3 (40 μm)	90.0	2.5	2.8
P5	L3 (40 μm)	60.0	2.5	1.8
P6	L3 (40 μm)	60.0	2.5	3.8

## Data Availability

The original contributions presented in this study are included in the article. Further inquiries can be directed to the corresponding author.

## Abbreviations

The following abbreviations are used in this manuscript:

UAVs	Unmanned aerial vehicles
LWA	Leaf wall area
TRV	Tree row volume
PI	Penetration index
D_V0.5_	Volume median diameter
LSD	Least significant difference
